# Use of a Waste-Derived Linde Type-A Immobilized in Agarose for the Remediation of Water Impacted by Coal Acid Mine Drainage at Pilot Scale

**DOI:** 10.3390/ma16114038

**Published:** 2023-05-29

**Authors:** Cristiano Luiz Chostak, Aurora López-Delgado, Isabel Padilla, Flávio Rubens Lapolli, María Ángeles Lobo-Recio

**Affiliations:** 1 Department of Sanitary and Environmental Engineering, Federal University of Santa Catarina, Florianópolis 88040-900, SC, Brazil; f.lapolli@ufsc.br (F.R.L.); maria.lobo@ufsc.br (M.Á.L.-R.); 2 Academic Department of Language, Technology, Education and Science, Federal Institute of Santa Catarina, Florianópolis 88020-300, SC, Brazil; 3 Eduardo Torroja Institute for Construction Sciences, IETcc, CSIC, 28033 Madrid, Spain; alopezdelgado@ietcc.csic.es (A.L.-D.); isabel.padilla@ietcc.csic.es (I.P.); 4 Department of Energy and Sustainability, Federal University of Santa Catarina, Araranguá 88906-072, SC, Brazil

**Keywords:** industrial waste, zeolitic material, immobilization, agarose gel, remediation, mine-impacted water

## Abstract

A new adsorbent based on an immobilized waste-derived LTA zeolite in agarose (AG) has proven to be an innovative and efficient alternative for removing metallic contaminants from water impacted by acid mine drainage (AMD) because the immobilization prevents the solubilization of the zeolite in acidic media and eases its separation from the adsorbed solution. A pilot device was developed containing slices of the sorbent material [AG (1.5%)–LTA (8%)] to be used in a treatment system under an upward continuous flow. High removals of Fe^2+^ (93.45%), Mn^2+^ (91.62%), and Al^3+^ (96.56%) were achieved, thus transforming river water heavily contaminated by metallic ions into water suitable for non-potable use for these parameters, according to Brazilian and/or FAO standards. Breakthrough curves were constructed and the corresponding maximum adsorption capacities (mg/g) (Fe^2+^, 17.42; Mn^2+^, 1.38; Al^3+^, 15.20) calculated from them. Thomas mathematical model was well fitted to the experimental data, indicating the participation of an ion-exchange mechanism in the removal of the metallic ions. The pilot-scale process studied, in addition to being highly efficient in removing metal ions at toxic levels in AMD-impacted water, is linked to the sustainability and circular economy concepts, due to the use as an adsorbent of a synthetic zeolite derived from a hazardous aluminum waste.

## 1. Introduction

The extraction of natural mineral resources, such as coal mining, has been a long-standing cause of environmental imbalance in ecosystems. It is responsible for damaging soil and water besides impairing the survival of different species living in impacted environments. One of the main reasons is the release of acid mine drainage (AMD) to freshwater from exposing slag from mining (pyrite-rich rocks) to weathering, oxidizing agents, and water, which in turn produces acidic aqueous leachate that are toxic and rich in dissolved metals (Fe, Al, Mn, etc.) and sulfates [1,2]. Water bodies impacted by AMD can have their physicochemical properties alternated drastically, such as high sulfate content, low pH (<4), and high concentration of metallic species (Fe^2+^, Mn^2+^, Al^3+^) [2,3].

From the above, it is expected that mining operations play a significant role in the generation of AMD [4]. In China, for instance, producing 1 ton of coal generates around 2.1 tons of AMD [5]. As a result of that, thousands of kilometers of AMD-polluted rivers and streams alike are reported in several parts of the world [5,6]. Some of these highly polluted freshwater bodies end up transporting tons of dissolved metals and discharging them into oceans [7]. For example, Olías et al. [8] estimated the Odiel and Tinto rivers (southwestern Spain) transport and release to the Gulf of Cádiz approximately 7.9 tons of iron, 5.8 tons of aluminum, and 1.6 tons of manganese per year.

In Brazil, more specifically its southern part, the coal basin of Santa Catarina (SC) State is known to be heavily affected by AMD. There are several official reports on low pH, and high electric conductivity and levels of metallic ions (Fe, Al, Mn) and sulfates in the Sangão river (Forquilhinha-SC) [3,9,10,11,12]. The measured levels were well above the maximum allowed by national and international standards [13,14,15,16]. As an aggravating factor, metal ions are non-biodegradable contaminants capable of bioaccumulating and causing irreversible damage to the local biota and human populations living nearby AMD-impacted rivers [17,18,19,20]. Consequently, the water resources in the region are often found unfit for both human consumption and secondary purposes, such as agricultural irrigation. Due to these reasons, numerous studies have sought to improve and/or develop technologies for treating contaminated waters in the region, striving to remove metallic contaminants to achieve at least non-drinking use purposes [10,11,21,22,23]. 

Sorption is a technique that has been widely explored in the environmental remediation of AMD-impacted water. The main reasons are its simplicity, relatively low cost, high-quality effluent, low waste generation (e.g., toxic sludge), and great number of low-cost alternative adsorbents available for this technique [3,9,12,24]. Zeolites, both natural and synthetic, stand out among the adsorbents frequently used to remove metallic contaminants. The sorption capacity of these materials is well known and attributed to its structural and morphological characteristics [25,26]. Moreover, the ability to synthetize zeolite materials has expanded the possibilities of using these adsorbents [27,28,29]. This is the case for the Linde type-A zeolite (LTA) used in this study, synthesized from hazardous waste from the aluminum industry [30,31]. LTA is formed by a microporous crystalline structure with internal channels of negative net charge. This allows cation exchange with metallic contaminants, in addition to electrostatic sorption [29,30,32].

The above-mentioned LTA properties have been demonstrated in a study by experiments aiming at the removal of metallic contaminants from a synthetic solution alike AMD-impacted water [24]. In our previous research, we discussed the LTA immobilization in agarose gel (AG), which was successfully applied in batch regime for removal of Fe^2+^, Mn^2+^, and Al^3+^ ions from a synthetic solution and real samples of AMD-impacted water [33,34]. The goal was achieving zeolite stability under acidic conditions (characteristic of AMD-impacted water) as well as facilitate the removal of the ion-saturated adsorbent from the treated medium. In the present work, the behavior of the LTA zeolite immobilized in agarose gel is studied for the removal of Fe^2+^, Mn^2+^, and Al^3+^ ions from AMD-impacted water in continuous upward flow. The remediation experiment includes the LTA immobilization in agarose gel and the pilot-scale equipment used to apply the treatment with the immobilized LTA to the AMD-impacted water from Sangão river (southern Santa Catarina). Its efficiency of the treatment was verified through data treatment via breakthrough curves and data fitting to mathematical models.

## 2. Materials and Methods

### 2.1. Materials, Reagents, and Analytical Methods

The LTA zeolite was synthetized and characterized by López-Delgado et al. [31]. The agarose was acquired from Sigma-Aldrich, and it shows the following specifications: type I, polymerization temperature of 36 ± 1.5 °C (1.5% gel); strength (1.5%, *m*/*v*) > 2500 g/cm^2^; and fusion temperature of 87 ± 1.5 °C. The prototype equipment used for pilot-scale experiments in continuous flow (dimensions available in section item 2.3) was built using recycled materials based on polymers (PVC—polyvinyl chloride; and PE—polyethylene) in addition to 3D digital printing (Creality Ender3-V2 model) of ABS (Acrylonitrile Butadiene Styrene) filaments.

The AMD-impacted water used in the continuous upward flow studies was collected from the Sangão river (Forquilhinha/SC, in the coal region of Santa Catarina in southern Brazil, coordinates 28°45′38.2″ S 49°25′56.5″ W). The collected samples were stored in polypropylene bottles and kept at 4 °C, filtered and characterized on the same day the collection took place in order to determine the pH, conductivity, turbidity, and concentration of Fe^2+^, Mn^2+^, and Al^3+^ metal ions. In all experiments, the metal ion concentration was determined by UV-VIS spectroscopy on an Agilent Cary 60 Spectrophotometer, using the adapted methods of Eriochrome Cyanine R for the complexation of Al (λ = 535 nm), Phenanthroline for the complexation of Fe (λ = 510 nm), and Periodate for the oxidation of Mn in permanganate (λ = 525) [15,35,36]. The pH was measured with a bench pHmeter (Thermo Fisher Scientific, Scientiphic Orion 3Stars), electric conductivity with a micro-processed bench conductivity meter Bel W12D, and turbidity monitored with a digital turbidimeter (ASKO TU 430). The sulfate concentration was determined with the sulfaver method (Spectrophotometer HACH DR 5000) [35].

### 2.2. Preparing the Agarose-Immobilized LTA Zeolite

In order to be used as an adsorbent, the LTA zeolite was washed three times with ultrapure water, vacuum filtered through a 0.45-µm cellulose acetate membrane, dried afterwards in an oven at 80 °C for 24 h, and then kept within a desiccator [33]. 

**Figure 1:**
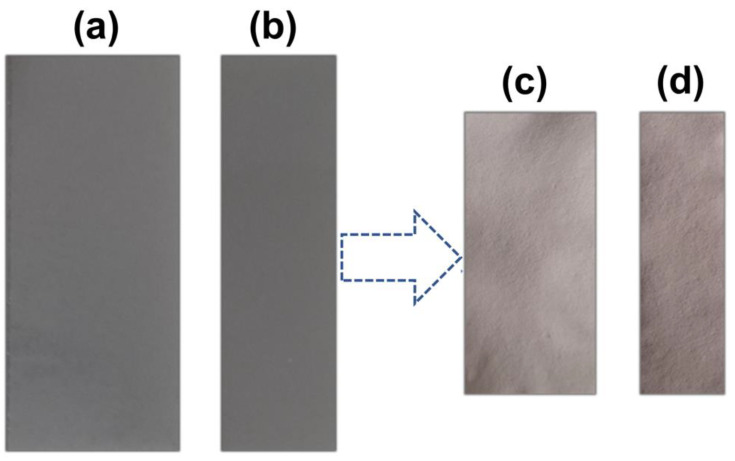
Film cut into slices (**a**) 4.1 cm × 9.5 cm and (**b**) 2.7 cm × 9.5 cm; dry film (**c**) 3.1 cm × 6.5 cm and (**d**) 2.0 cm × 6.5 cm.

The preparation of the adsorbent material was previously studied and optimized [33]. Basically, the LTA zeolite immobilized in agarose gel was prepared from a suspension of LTA (8%, *m*/*v*) and agarose (1.5%, *m*/*v*) in ultrapure water heated at 90 ± 1 °C and stirred until suspension homogenization. This homogeneous suspension was then cooled to 55 ± 1 °C, and the gel was molded by pouring 30 mL between two glass plates, which were previously heated (~60 °C) and separated with 0.92-mm spacers to delimit thickness of the formed film [33,37,38]. The produced films were then cut into slices (Figure 1a,b) and dried at room temperature for 72 h (Figure 1c,d).

In a previous study [33], AG-LTA was characterized, before and after sorption of metal ions, via scanning electron microscope (SEM), energy dispersive X-ray (EDS), X-ray diffraction (XRD), Fourier transform infrared spectroscopy (FT-IR), and thermogravimetric analyses (TGA). That study showed no alterations in the structure of LTA zeolite after immobilization.

### 2.3. Construction and Experimental Design of the Pilot-Scale System

**Figure 2:**
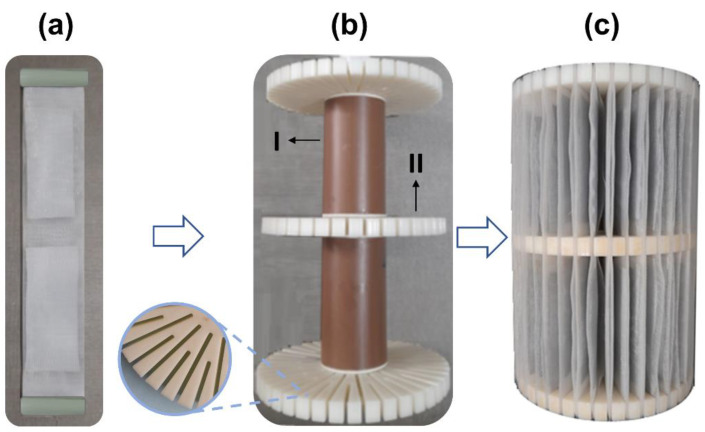
Support built for using AG-LTA for the pilot-scale experiment: (**a**) adsorbent between two polyethylene screens; (**b**) main support in PVC and ABS (diameter 11.5 cm; height 18 cm); (**c**) completed support with the adsorbent.

Viewing a scale-pilot treatment system for AMD-impacted water, a device was built for accommodate the AG-LTA adsorbent consisting in an apparatus formed by the junction of two 50-mesh polyethylene screens (Figure 2a) for allocating the adsorbent; a 18-cm tall main support (Figure 2b) formed by a PVC central axis (I); and three 3D-printed ABS circular pieces (II) with diameter of 11.5 cm, and cuts in sections of 3.8 cm and 2.5 cm for fixing the screens with the adsorbent. Figure 2c illustrates the main support completed with the adsorbent (AG-LTA).

**Figure 3:**
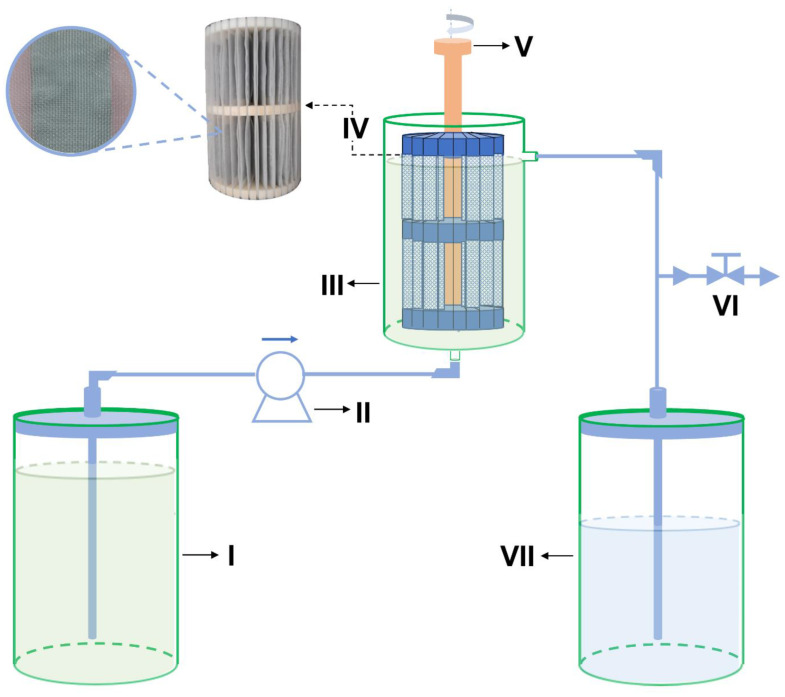
Scheme for pilot-scale treatment of AMD-impacted water: (I) and (VII) reservoirs; (II) peristaltic pump; (III) reactor; (IV) support with AG-LTA adsorbent; (V) stirring device; (VI) sample collection valve.

The treatment works by allowing ascending continuous flow (Figure 3) in a system consisting of two reservoirs with capacity of 5 L (I and VII) for influent and effluent amounts, respectively; a peristaltic pump (II); a PVC reactor (III) with useful volume during operation of ~2.5 L; a main device (IV) containing the adsorbent (AG-LTA) between polyethylene screens; a stirring device (V); and a sample collection valve (VI).

### 2.4. Operating Conditions for Adsorbent Application

The experiment was conducted in triplicate at a temperature of 24.0 ± 1.0 °C, with samples of AMD-impacted water containing metallic ions (Fe^2+^, Mn^2+^, and Al^3+^). The mass of LTA immobilized in agarose gel used in the support was 31.19 g. The reactor (2.5 L) was fed with a 10.4 mL/min flow rate and the system operated for a period of 4320 min under stirring rate 60 rpm. The operating parameters of the pilot were defined in a preliminary study with a synthetic solution similar to the water impacted by DAM. Equation (1) described the hydraulic retention time (HRT):

(1) $$HRT=\frac{V_{r}}{Q}$$

where *V_r_* is the useful volume of the reactor in operation (L), and *Q* is the flow rate (L/min).

During the experiment, samples were collected at predetermined periods to monitor the pH, turbidity, and concentration of metallic ions (C_t_) at the time t and the adsorbent saturation (AG-LTA). 

### 2.5. Breakthrough Curves for the Pilot-Scale System

To evaluate the behavior of ion metal removal, as well as to estimate the maximum sorption capacity (q_max_) of AG-LTA during a continuous flow, breakthrough curves were constructed. The breakthrough curve can be expressed in terms of adsorbed metallic ion concentration (C_ads_ = *C*_0_ − *C*_t_) or the normalized concentration, as *C*_t_*/C*_0_ in function of the effluent volume (V_ef_) or of the time (t) [39]. The effluent volume (V_ef_), or the adsorbate solution treated volume, can be calculated by using Equation (2):

(2) $$V_{ef}=Qt$$

where *Q* is the flow rate (mL/min) and *t* time (min), respectively. 

The area (A) under the breakthrough curve is estimated using Equation (3), being the saturation time considered in this study corresponding to C_t_/C_0_ ≈ 0.99. From Equation (4), the total mass (m_total_) adsorbed by the AG-LTA for each metal ion in the influent is thus determined. The maximum sorption capacity (q_max_) is determined from applying Equation (5) [39,40].

(3) $$A=\int_{t=0}^{ts}\left(1-\frac{C_{t}}{C_{0}}\right)dt$$

(4) $$m_{total}=\frac{QC_{0}}{1000}A$$

(5) $$q_{max}=\frac{m_{total}}{W}$$

where *m_total_* is the total mass (mg) of each ion adsorbed in the device until saturation time; *Q* is the flow rate (mL/min); *t_s_* is the saturation time (min); *C*_0_ and *C_t_* are the ion concentrations (mg/L) in the influent and effluent, respectively; *W* is the adsorbent mass (g); and *q_max_* is the maximum sorption capacity (mg/g).

Equation (6) was used to calculate total mass (M_total_) of each ion passing through the column at time t (for instance, t_breakthrough_ and/or t_saturation_), in order to determine the removal percentage with Equation (7):

(6) $$M_{total}=\frac{QC_{0}t}{1000}$$

(7) $$Removal\;(\%)=\frac{M_{total}}{m_{total}}100$$

where *M_total_* is the total mass (mg) of the ion that passed through the column; *Q* is the flow rate (mL/min); t is the time (min); *C*_0_ is the ion concentration (mg/L) in the influent; and m_total_ is the total ion mass (mg) adsorbed in AG-LTA (obtained by Equation (4)).

### 2.6. Mathematical Models for Breakthrough Curves

Aiming at assessing the model that best describes the breakthrough curves and verifying the accuracy of the experimental q_max_ values, the obtained data were confronted with the models of Thomas and Yan. As they require little input (e.g., operational variables), these models have been widely used to describe results from similar experiments as they do not require, for instance, the knowledge of bed void fraction, adsorbent density, or bed length [41,42,43]. Originally developed to describe ion exchange at liquid–solid interfaces, the Thomas model is now widely used to evaluate the sorption performance in columns [44,45]. In addition to predicting breakthrough curves, it allows to determinate the maximum theoretical sorption capacity (q_max_) [40]. Mathematically, this model can be expressed through Equation (8) [45]:

(8) $$\frac{C_{t}}{C_{0}}=\frac{1}{1+exp(a-bt)}$$

$$a=\frac{k_{TH}q_{TH}W}{Q};b=k_{T}C_{0}$$

where the terms *k_TH_* (L/mg∙min), *q_TH_* (mg/g), *Q* (L/min), t (min), and *W* (g) are the Thomas model constant, maximum sorption capacity, flow rate, flow time, and the adsorbent mass, respectively. *C*_0_ and *C_t_* are ion concentrations (mg/L) in the influent and effluent, respectively. 

Alternatively, the Yan model is an empirical relationship. It was proposed to minimize possible errors resulting from using the Thomas model, mainly when operating times are extremely long or short [40,46]. The model, in addition to accurately describing the breakthrough curves, also provides the maximum sorption capacity [42]. The mathematical expression of Yan model is the following [39]:

(9) $$\frac{C_{t}}{C_{0}}=1-\frac{1}{1+{\left(\frac{Qt}{b}\right)}^{a}}$$

$$a=\frac{k_{y}C_{0}}{Q};b=\frac{q_{y}W}{C_{0}}$$

where the terms *k_y_* (L/mg∙min), *q_y_* (mg/g), *Q* (L/min), *t* (min), and *W* (g) are the Yan model constant, maximum sorption capacity, flow rate, flow time, and the adsorbent mass, respectively. *C*_0_ and *C_t_* are ion concentrations (mg/L) in the influent and effluent, respectively.

The equation parameters of the respective abovementioned models were obtained via non-linear regression. The model that best described the experimental data was defined based on the following error functions: determination coefficient (*R*^2^); sum of squares of the error (SSE); and chi-square coefficient (*χ*^2^). The *R*^2^, *χ*^2^, and SSE mathematical expressions are shown below [43,47,48]:

(10) $$R^{2}=\frac{\sum_{i=1}^{n}(y_{cal}-\bar{y_{exp}}{)}^{2}}{\sum_{i=1}^{n}(y_{cal}-\bar{y_{exp}}{)}^{2}+\sum_{i=1}^{n}(y_{cal}-y_{exp}{)}^{2}}$$

(11) $$SQE=\sum_{i=1}^{n}(y_{cal}-y_{exp}{)}^{2}$$

(12) $$\chi^{2}=\sum_{i=1}^{n}\left(\frac{{(y}_{cal}-y_{exp}{)}^{2}}{y_{cal}}\right)$$

where *y_exp_* is the experimental dependent variable, *y_cal_* the dependent variable calculated by the theoretical model, and *n* the number of trial points.

## 3. Results and Discussion

### 3.1. Characterization of the AMD-Impacted Water

As shown in Table 1, water samples from the Sangão river have physicochemical characteristics common to aqueous media impacted by AMD such as a high concentration of metallic ions and sulfate; low pH; and high electric conductivity [3,9,10,11]. These parameters indicate the water quality does not meet the Brazilian standard regarding class III water (suitable for non-potable secondary use) which establishes 0.2 mg/L (Al^3+^); 5.0 mg/L (Fe^2+^); 0.5 mg/L (Mn^2+^); and pH 6–9 [13]. The same can be said about the guidelines established by the Food and Agriculture Organization (FAO of the United Nations for water destiny to irrigation, which are the following: 5.0 mg/L (Al^3+^); 5.0 mg/L (Fe^2+^); 0.2 mg/L (Mn^2+^); and pH 6–8 [14,15].

Previous studies conducted with water abstracted from the same region indicated similar or higher concentration values to this study. Rodrigues et al. [11] reported concentrations of 18.4–54.8 mg/L for iron ions, 14.32–24.6 mg/L for aluminum ions, and 2.0–3.2 mg/L for manganese ions. Núñez-Gómez et al. [9] found concentrations (in mg/L) of 56.30 for iron ions, 35.92 for aluminum ions, and 2.72 for manganese ions. These results reinforce the need to control the environmental impacts derived from mining operation and of the search for alternatives for remediation of AMD-impacted water in the region.

### 3.2. Operationalization and Breakthrough Curves

**Figure 4:**
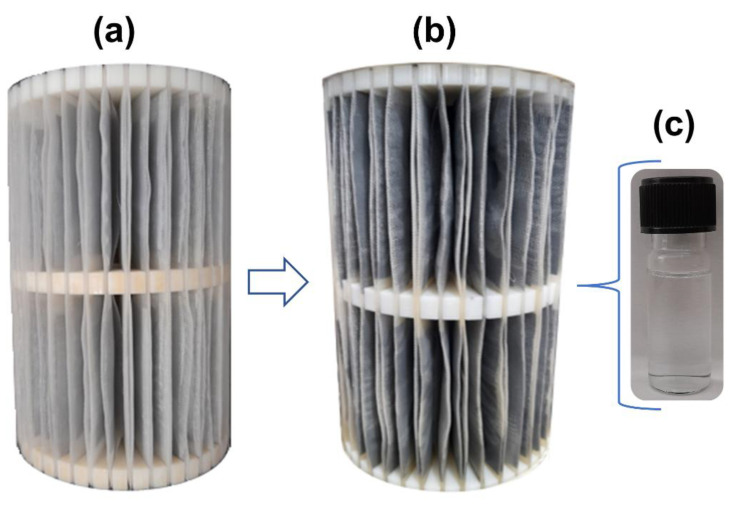
Support with AG-LTA: (**a**) before experiments; (**b**) after experiments for remediation of AMD-impacted water; (**c**) sample collected during the operation that typifies the treated effluent.

As mentioned above, the pilot system was operated with a constant up flow rate (10.4 mL/min) and hydraulic retention time (HRT) of 240 min (Equation (1)). Figure 4a illustrates the completed adsorbent support and Figure 4b shows the same support after 4320 min of use in the remediation of the AMD-impacted water. Notably, the system proposed was robust enough to treat AMD, including the AG-LTA adsorbent material exposed to acidic conditions (pH < 4) for an extended period, confirming its expected stability in this medium. Furthermore, the support’s physical aspect, the absence of precipitates or suspended material in the reservoir, and the low turbidity values (<0.01 NTU) observed in the collected samples (Figure 4c) corroborate to the robustness of the system. It is worth emphasizing that powdered LTA zeolite is soluble when the pH is below 4. However, this problem is solved by incorporating it into the agarose gel, enabling its utilization as a sorbent even at low pH levels and eliminating the challenges associated with separating the adsorbent from the sorbate solution through filtration or centrifugation [33].

**Figure 5:**
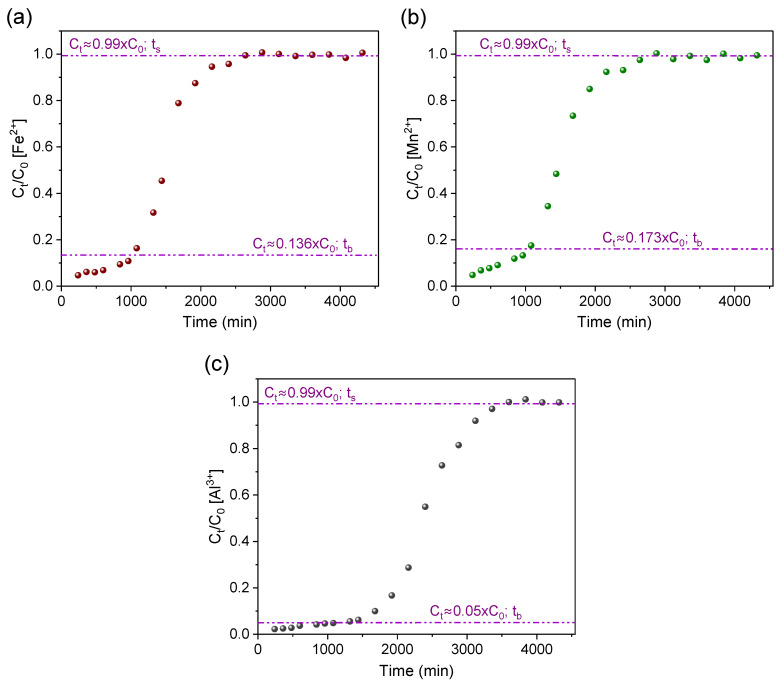
Breakthrough curves for studying the removal of (**a**) iron, (**b**) manganese, and (**c**) aluminum ions with AG-LTA from AMD-impacted water in a pilot system (t_b_ and t_s_ are the adsorbent breakthrough and saturation times, respectively).

Figure 5 illustrates the breakthrough curves estimated from the normalization of metal ion concentrations (C_t_/C_0_) over the total operating time of the experiments. The results are summarized in Table 2 and commented on in the next paragraphs.

Figure 5a,b show the breakthrough curves for Fe^2+^ and Mn^2+^ ions, respectively. For both metals, it was adopted as breakthrough time (t_b_) the moment when the Mn^2+^ and Fe^2+^ ions concentration (C_t_) were, respectively, in accordance with maximum values permitted by Brazilian standards for class III water [13]. Thus, the breakthrough time (t_b_) for the Fe^2+^ ion (Figure 5a) was established as ~1027 min (C_t_ ≈ 0.136 × C_0_ ≈ 5.0 mg/L), meaning that during this period the treatment maintains sufficient removal efficiency to reduce (to about 86–95%) the iron ion concentration to values that meet Brazilian and FAO standards [14]. It is important to note the high removal percentage (93.45%) for the iron ion determined until the breakthrough time (Table 2). 

The saturation of the adsorbent AG-LTA with iron ion was observed after a period of 2880 min (t_s_). The removal percentage at saturation point was determined to be 49.67% and the maximum experimental sorption capacity (q_max_) estimated at 17.42 mg/g_LTA_ (Table 2). The literature reports results for iron ion removal with alternative adsorbents comparable to this study (Table 3). For instance, Núñez-Gómez et al. [2] reported an adsorption capacity in continuous flow of 17.43 mg of iron ions per g of shrimp shell, and the study by Li et al. [49] found a maximum removal of 18.519 mg of Fe^2+^ per gram of ashes from domestic waste.

The Mn^2+^ ion breakthrough curve (Figure 5b) behaved similarly to that observed for Fe^2+^ ion. However, the Mn^2+^ ion breakthrough time (t_b_) was observed in ~1080 min (C_t_ = 0.173 × C_0_ ≈ 0.5 mg/L), a period in which the treatment maintained sufficient removal efficiency to decrease the Mn^2+^ ion concentration (in about 86–95%) and meet levels that would still comply with Brazilian standards [13]. It is important to note that during the first 360 min, the treatment could reduce manganese ion concentration (C_t_ = 0.069 × C_0_ ≈ 0.2 mg/L) to meet FAO guidelines [14,15]. The results available in Table 2 corroborate with these findings since the removal percentage determined in the period until reaching the breakthrough time was relatively high (91.62%).

The removal efficiency was lower for Mn^2+^ when compared to Fe^2+^ until near the saturation point of the adsorbent, in which an inversion in this trend occurred shown by the removal percentages (Table 2). This is due to the large availability of active sites in the zeolite at the beginning of the experiments. This happens because iron has an advantage over manganese ion, which has the same charge (2+) at a lower initial concentration (2.88 mg/L); with the availability decrease in the zeolite active sites, Mn^2+^ adsorption tends to increase in relation to iron, since the hydrated radius of Mn^2+^ is relatively smaller, which facilitates sorption through the microporous structure of the agarose-immobilized LTA zeolite [24,50]. The estimated maximum sorption capacity achieved at saturation time (t_s_ ≈ 2880 min) was 1.38 mg Mn^2+^/g LTA. Similar values to this work are found in the literature (Table 3), e.g., 0.498 mg of Mn^2+^ per gram of ashes from domestic waste [49] and 0.076 mg and 0.52 mg of Mn^2+^ per gram of two different natural zeolites [51,52].

A significant reduction in concentration (75–98%) for the Al^3+^ ion was observed during the experiments (Figure 5c) and this lasted for a considerable period (~2125 min) of operation (C_t_ = 0.255 × C_0_), and it was sufficient to meet FAO’s guideline. However, the removals were still not high enough to comply with Brazilian standards for class III water. Therefore, the curve point where the adsorbate effluent concentration (C_t_) was 5% of the influent concentration (C_t_ = 0.05 × C_0_) was adopted as the breakthrough time (t_b_ ≈ 1320 min); Thus, the Al^3+^ ion removal percentage (96.56%) was relatively higher when compared to the other metal ions (Table 2). This was observed throughout the experiments and confirmed by the aluminum ion removal percentage (64.77%) in the saturation period, which was slightly higher in relation to Fe^2+^ and Mn^2+^ ions. These observations indicate a high affinity of the agarose-immobilized LTA zeolite for aluminum ions. This behavior is attributed to the high positive charge density of Al^3+^ and its small effective ionic radius (0.535Å) [53] which favor its attraction to the oxygenated and negative LTA surface and its penetration into zeolite pores and channels [24].

The maximum sorption capacity attributed to aluminum was 15.20 mg/g_LTA_ (Table 2), estimated based on the time required (3600 min) to saturate the adsorbent. This value, besides being consistent with a recent report of Al^3+^ ions removal with powdered LTA zeolite (13.93 mg/g) [24], is comparable to other literature findings (Table 3). For example, a maximum sorption of 4.37 mg of Al^3+^ per gram of a modified granular activated carbon [54], and the maximum removal of 5.831 mg of Al^3+^ per gram of activated carbon from date palm waste [55]. Considering that the sorption experiment was conducted with river water containing the three ion types competing among themselves, the maximum total capacity of adsorption by the zeolite was 34.00 mg/g_LTA_, a value regarded as promising for the treatment of AMD-impacted water on larger scales. 

### 3.3. pH Variation during Operation

**Figure 6:**
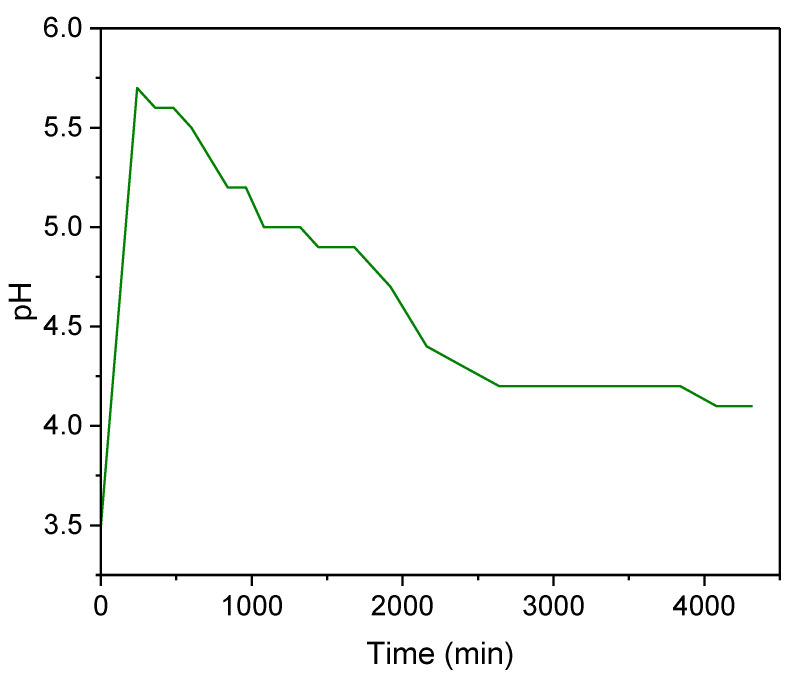
pH variation during pilot-scale experiments for the treatment of AMD-impacted water with AG-LTA.

Figure 6 illustrates the results for pH monitoring during the experiments. During the initial stage of the treatment (240–1080 min), there is a fast rise in pH to 5.7. Subsequently, there is a gradual decrease throughout the remaining duration of the treatment, eventually stabilizing at around 4.1 (when the adsorbent becomes saturated). This initial behavior can be explained by the cationic exchange between protons of the strong acid medium and the zeolite exchanger ions (Na^+^, K^+^) [52,56], followed by the displacement of these protons on the zeolite by the metallic ions (with greater charge) from the AMD-impacted water, thus contributing to pH reduction. This pH behavior was also observed in a previous study achieving AG-LTA saturation in batches during the treatment of AMD-impacted water [33]. However, in the present study, the initial pH increase was greater (5.7) in comparison with previous studies with AG-LTA, which is justifiable enough due to the higher adsorbent load used.

During the experiments, cation exchange availability at the zeolite’s active sites decreases together with the pH, and therefore adsorption processes due to multilayer electrostatic effects prevail, as reported in our previous study [34]. It is important to mention the increased pH in the experiments of this study was not sufficient to reach the precipitation pH of Mn and Fe ions as hydroxides in the solution (8.0 and 10.0 for Fe^2+^ and Mn^2+^, respectively, in concentrations of ~10^−3^ mol/L) [57,58]. Nonetheless, Al ion precipitation on the surface of the adsorbent cannot be ruled out at initial stages since the initial pH was above 5.0 (pH of hydroxide precipitation) [59]. A similar behavior was observed in the recent literature with powdered LTA zeolite for the Al^3+^ ion removal [24]. However, in the present study, the removal via sorption/cation exchange prevails since significant decreases in the aluminum ion concentration occur at pH below 5. For example, for t = 1440 min, pH = 4.9 (Figure 6), and C_t_ = 0.06 × C_o_ = 1.17 mg/L (Figure 5c), indicating an Al^3+^ reduction of ~93%.

### 3.4. Applied Mathematical Models

**Figure 7:**
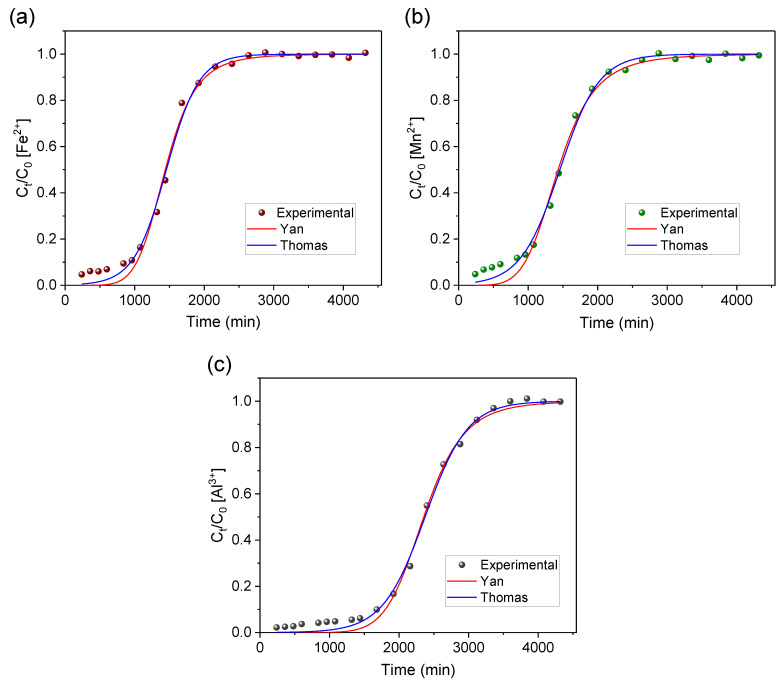
Experimental data fitting to the Yan and Thomas models for studying the removal of (**a**) iron, (**b**) manganese, and (**c**) aluminum ions from AMD-impacted water with AG-LTA in a pilot system.

The experimental data obtained from the breakthrough curves were evaluated by applying the mathematical models of Thomas and Yan (Equations (8) and (9)) in order to further interpret the findings. Figure 7 and Table 4 display the results for the non-linear regression.

Both models were suited (R^2^ > 0.99) to describe the experimental data obtained in the study on the removal of metal ions from AMD-impacted water (Table 4). Nevertheless, by considering the R^2^ value (Fe^2+^, 0.9952; Mn^2+^, 0.9953; Al^3+^, 0.9966) and the lowest values for the other error functions, the best fit for all metals was in the Thomas model. This is in line with the model’s principle of describing processes involving ionic exchanges in sorption columns [44]. These results agree with our previous isothermal and kinetic study, which indicated a chemisorption/ion exchange sorption mechanism [34]. LTA zeolite has a high cation exchange capacity [30] which was already widely explored in previous sorption studies, especially in the removal of metallic contaminants [24,34]. The maximum sorption capacities (q_TH_) estimated by the Thomas model (Table 4) for agarose-immobilized LTA were 17.75 mg/g, 1.40 mg/g, and 15.48 mg/g for Fe^2+^, Mn^2+^, and Al^3+^, respectively. These values (q_TH_) are close to q_max_ values obtained via area integral (Table 2) for the metallic ions Fe^2+^ (17.42 mg/g), Mn^2+^ (1.38 mg/g), and Al^3+^ (15.20 mg/g), thus showing the accuracy of the mathematical model of Thomas to predict maximum sorption capacities. 

Although the Yan model may not be the most accurate in describing the experimental data based on the error functions, it yielded maximum sorption capacities of 17.54 mg/g, 1.37 mg/g, and 15.35 mg/g for Fe^2+^, Mn^2+^, and Al^3+^, respectively. These values closely align with those predicted by the Thomas model and obtained through area integrals.

When considering the agarose-immobilized LTA zeolite obtained through the recovery of toxic aluminum residues at a relatively low cost [31], it is noteworthy that the maximum sorption capacities determined through integration of the area and confirmed by mathematical models are comparable to literature findings for other adsorbents, such as activated carbon [49,51,54,55,60]. Moreover, the pilot-scale study results suggested the possibility of a future application of the treatment on a larger scale with several devices in series. This would allow to increase removal rates while complying with Brazilian and international standards. Further studies will be conducted by the authors in this regard, striving to reuse the adsorbent and recover the metallic ions adsorbed in the material.

## 4. Conclusions

The AG-LTA material maintained the excellent adsorbent properties of LTA zeolite. Furthermore, it demonstrated two important advantages when compared to powdered zeolite: stability in strongly acidic media, and easy separation from the medium once saturated with Fe^2+^, Al^3+^, and Mn^2+^ ions, thus avoiding complex filtration or centrifugation steps. 

The pilot-scale device containing the AG-LTA together with the experiments conducted in continuous flow showed that an efficient removal of these ions from AMD-affected water can be achieved. 

The results showed that the obtained removal rates are high enough to comply with the FAO’s guideline for Al^3+^ ion and the most restrictive Brazilian laws for Fe^2+^ and Mn^2+^ ions. The removal of ions was mainly via sorption/cation exchange, although some precipitation such as hydroxide for aluminum cannot be ruled out. The treatment of the data obtained from breakthrough curves suggested that the experimental data fit well to the Thomas model, confirming the existence of ion exchange between the Na^+^/K^+^ zeolitic cations and the metallic ions from AMD-impacted water. Maximum sorption capacities obtained through area integral were very close to those from the Thomas model, thus evidencing the accuracy of this mathematical model. 

It is to note that the pilot-scale treatment system of AMD-impacted water with the AG-LTA sorbent, in addition to the high efficiency in removing metallic ions at toxic level, presents aspects of sustainability and circular economy principles. This is evident in, for example, the use of a synthetic zeolite derived from a hazardous aluminum waste. 

The findings of this study support the feasibility of implementing the developed system in a serial configuration. This approach would enhance the removal rates and increase the potential for meeting stringent environmental standards. As a result, it would be possible to transform polluted river water into water suitable for non-potable use, hence preserving the scarce sources of quality water for drinking use in coal mining regions.

## Figures and Tables

**Table 1:** Characteristics of the filtered AMD-impacted water.

Metal	C_0_ (mg/L)	pH	Conductivity (µS/cm)	Sulfate (mg/L)
Fe	36.51 ± 0.54	3.5 ± 0.1	858 ± 5	416 ± 4
Al	19.55 ± 0.32			
Mn	2.88 ± 0.17			

OBD_5_, OCD, and non-purgeable organic carbon were negligible.

**Table 2:** Data obtained from breakthrough curves in the breakthrough and saturation times of AG-LTA (31.19 g) under constant flow rate (10.4 mL/min) and hydraulic detention time (240 min).

Metal	Breakthrough Point				Saturation Point				
	t_b_ (min)	m_total_ (mg)	M_total_ (mg)	R (%)	t_s_ (min)	m_total_ (mg)	M_total_ (mg)	R (%)	q_max_ (mg/g)
Fe	1027	364.41	389.96	93.45	2880	543.19	1093.55	49.67	17.42
Mn	1080	1529.64	32.35	91.62	2880	43.13	86.26	50.00	1.38
Al	1320	259.15	268.38	96.56	3600	474.10	731.95	64.77	15.20

t_b_ = breakthrough time; t_s_ = saturation time; M_total_ = total mass of the íon passing through the column; m_total_ = total mass of the adsorbed ion; R (%) = total removal percentage in t_b_ or t_s_; q_max_ = maximum capacity of experimental sorption (mg/g_LTA_).

**Table 3:** Maximum adsorption capacity of some sorbents for the metal ions Fe^2+^, Mn^2+^, and Al^3+^.

**Adsorbent**	**Adsorbate**	**Adsorption Capacity (mg/g)**	Ref.
Shrimp shell	Fe^2+^	17.43	[2]
Domestic waste Ash	Fe^2+^	18.519	[49]
	Mn^2+^	0.498	[49]
Natural zeolite	Mn^2+^	0.076	[50,51]
Natural zeolite	Mn^2+^	0.52	[50,51]
Modified activated carbon	Al^3+^	4.37	[52]
Activated carbon date palm waste	Al^3+^	5.831	[53]
Powdered LTA zeolite	Al^3+^	13.93	[24]
AG-LTA	Fe^2+^	17.42	This study
	Mn^2+^	1.38	
	Al^3+^	15.20	

**Table 4:** Main parameters and error functions for fitting experimental data to the Thomas and Yan models. Experimental conditions: flow rate (Q) 10.4 mL/min; (mLTA) 31.19 g (1.5% AG–8.0% LTA); temp. 24.0 ± 1.0 °C; HRT 240 min.

Metal	Thomas’ Model					Yan’s Model				
	K_TH_ (mL/mg∙min)	q_TH_ (mg/g)	R^2^	χ^2^	SSE	K_y_ (mL/mg∙min)	q_y_ (mg/g)	R^2^	χ^2^	SSE
Fe	0.1169	17.75	0.9952	0.0009	0.0173	1.8596	17.54	0.9918	0.0015	0.0293
Mn	1.2292	1.40	0.9953	0.0008	0.0158	19.4993	1.37	0.9908	0.0016	0.0305
Al	0.1673	15.48	0.9966	0.0006	0.0125	4.3623	15.35	0.9945	0.0011	0.0201

## Data Availability

Not applicable.
